# Time spent cycling, walking, running, standing and sedentary: a cross-sectional analysis of accelerometer-data from 1670 adults in the Copenhagen City Heart Study: Physical behaviours among 1670 Copenhageners

**DOI:** 10.1186/s12889-019-7679-z

**Published:** 2019-10-24

**Authors:** Melker Staffan Johansson, Mette Korshøj, Peter Schnohr, Jacob Louis Marott, Eva Irene Bossano Prescott, Karen Søgaard, Andreas Holtermann

**Affiliations:** 1 Musculoskeletal Disorders and Physical Workload, National Research Centre for the Working Environment, Lersø Parkallé 105, 2100 Copenhagen Ø, Denmark; 2 Department of Sports Science and Clinical Biomechanics, University of Southern Denmark, Campusvej 55, 5230 Odense M, Denmark; 3 The Copenhagen City Heart Study, Bispebjerg and Frederiksberg Hospital, Nordre Fasanvej 57, Hovedvejen indg. 5, st, 2000 Frederiksberg, Denmark; 4 Department of Cardiology, Bispebjerg and Frederiksberg Hospital, Bispebjerg Bakke 23, 2400 Copenhagen, NV Denmark

**Keywords:** Cross-sectional study, General population, Adults, Older adults, Physical activity, Sedentary behaviour, Stationary behaviours, Accelerometer

## Abstract

**Background:**

Information about how much time adults spend cycling, walking and running can be used for planning and evaluating initiatives for active, healthy societies. The objectives of this study were to describe how much time adult Copenhageners cycle, walk, run, stand and spend sedentary using accelerometers, and to describe differences between population groups.

**Methods:**

In the fifth examination of the Copenhagen City Heart Study, 2335 individuals gave consent to wear accelerometers (skin-attached; right thigh and iliac crest; 24 h/day, 7 consecutive days) of which 1670 fulfilled our inclusion criteria (≥16 h/day for ≥5 days; median wear time: 23.8 h/day). Daily time spent cycling, walking, running, standing and sedentary was derived from accelerometer-based data using the Acti4 software, and differences between sex, age groups, level of education and BMI were investigated using Kruskal-Wallis rank sum tests.

**Results:**

Among those cycling (61%), the median cycling time was 8.3 min/day. The median time walking, running, standing and sedentary was 82.6, 0.1, 182.5 and 579.1 min/day, respectively. About 88% walked fast (i.e., ≥100 steps/min) ≥30 min/day. The shortest duration and lowest prevalence of cycling, walking and running were found among older individuals, those with a low level of education, and individuals being overweight or obese.

**Conclusions:**

We found a long duration and high prevalence of cycling and walking, but also that many adult Copenhageners spent much time sedentary. Population groups with low participation in physical activities such as cycling and walking should be targeted in future initiatives towards an active, healthy society.

## Background

Physical activity is essential for public health [1]. Activities such as cycling and walking are known to lower the risk of non-communicable diseases (NCDs) and mortality [2–7] while excessive sedentary behaviour has opposite effects on health [8–11]. Several countries and cities have therefore taken initiatives to increase physical activity in the population [12, 13]. For example, Copenhagen, Denmark, has improved bicycle infrastructure and walkability to promote active transportation by cycling and walking [12]. Evidently, cycling has increased in Copenhagen over the last two decades [14]. Further, both commuting and total cycling have been estimated to prevent substantial morbidity and mortality in Denmark [14]. Valid and reliable measurements of how much, how many and who that participate in physical *activities* such as cycling and walking are crucial to evaluate whether physical activity-promoting initiatives reach high-risk populations with low physical activity levels. Policy makers and other stakeholders can also use such information to plan and create active, healthy societies [1].

However, existing knowledge about how much time adults spend in different physical behaviours in urban cities like Copenhagen is limited, for several reasons. Firstly, most studies are based on self-reported data [3, 7, 15–17], which is prone to measurement error, recall and social desirability bias [18]. Secondly, the majority of accelerometer-based studies have focussed on intensity or energy expenditure by using counts per minutes [19–21]. This type of data does not convey information about the *type* of activity (e.g., cycling and walking) or the *body posture* (e.g., sitting and standing), and may be more challenging to communicate, understand and act upon for both researchers and stakeholders. Finally, some studies have used counting systems (i.e., number of cyclists and pedestrians) [14] that does not capture the duration of the activity or who that performs the activity (i.e., what population groups).

It is now possible to detect specific physical activity *types* such as cycling, running and walking, and *body postures* such as sitting and standing from thigh-based accelerometer data [22]. We believe that such information about physical activity types and stationary behaviours in different population groups can help both researchers, policy makers and other stakeholders to better identify, target and reach groups in need for preventive interventions.

Thus, the overall objectives of this study were to describe how much time Copenhageners spend cycling, walking, running, standing and sedentary, and to describe differences between population groups, using accelerometer-data from a large general population sample.

## Methods

### Study design and study population

#### Study design

This is a cross sectional analysis of data collected between October 2011 and February 2015 as part of the fifth examination of the Copenhagen City Heart Study (CCHS), a dynamic population-based cohort study [23].

#### Study population

In 1976, 19,329 individuals were randomly drawn from a source population and invited to participate in the *first* examination of the CCHS. The source population consisted of about 90,000 adults (≥20 years old) living in two parts of Copenhagen, Denmark. These were identified through the Copenhagen Population Register using a national registration number. Details about the source and initial study population are described elsewhere [23].

In the *fifth* examination, 9215 individuals from previous examinations (*n* = 8234) and from a new sample of younger subjects (*n* = 981) were invited to participate. The participants from previous examinations were invited regardless of whether they had moved to an address outside the study area or participated in previous examinations or not. Information about death and change of address was obtained from the Danish Civil Registration System.

Invitations were sent 3 weeks prior to a scheduled health examination and included a questionnaire and a pre-paid postcard where individuals could confirm their participation, change the appointment or decline to participate. In case of a non-returned postcard 1 week prior to the examination, a second invitation was sent. Non-responders and non-attenders at the day of the examination were sent a new invitation 6 months later.

### Data collection

#### Questionnaire

A self-administered questionnaire was used to collect data across a wide range of domains, including socioeconomic status; general, physical and mental health; symptoms and diseases; physical activity at work and leisure; tobacco and alcohol consumption; diet; medication use; health care-seeking behaviour; and familial disposition for cardiovascular and other NCDs. See Additional file 1 for an overview of the questions we used for the purpose of this study.

#### Physical examination

All participants underwent a physical examination at the test centre located at a public hospital in the Capital Region of Denmark. The study staff were trained in the examination procedures and had backgrounds as medical laboratory technicians, medical students or medical specialists.

The physical examination consisted of a variety of tests and measurements including a non-fasting venous blood sample. Details about the physical examination have been described elsewhere [23]. The tests relevant for this report were measurements of height and weight, waist and hip circumference and blood pressure (i.e., three consecutive blood pressure measurements were taken on the participants’ left arm using an electronic blood pressure monitor after five-minutes in a sitting position).

#### Accelerometer-based measurements of physical activity types and stationary behaviours

Data about physical activity types and stationary behaviours were collected using tri-axial accelerometers (ActiGraph GT3X+; ActiGraph, Pensacola, Florida, USA; sampling frequency: 30 Hz). As part of the physical examination, all participants were asked to wear two accelerometers 24 h a day for seven consecutive days. Consenting participants had one accelerometer attached on the anterior aspect of the right thigh midway between the greater trochanter and patella oriented along the axis of the thigh, and one accelerometer attached on the lateral aspect of the right iliac crest. The accelerometers were attached directly to the skin using a double-sided medical tape (Hair-Set for hairpieces; 3 M, Maplewood, Minnesota, USA) and wrapped with transparent adhesive film (OpSite Flexifix; Smith & Nephew, London, UK) to ensure a correct position during the measurement period.

Participants were provided with a diary to keep a record of their leisure time, work hours, time in bed, and any periods of non-wear time during the measurement period. They were also asked to make a *daily reference measurement* by standing still for 15 s and note the time in the diary. Finally, the participants were asked to only remove the accelerometers in case of adverse skin reactions, discomfort or pain, or affected sleep, and when going to a sauna. Participants were asked to return the accelerometers at the test centre or by mail using a pre-paid envelope. The accelerometers were initialised, and raw data was downloaded by study staff using the manufacturer’s software (ActiLife version 5).

This procedure has previously been used in studies validating the use of accelerometers to detect different physical activity types and stationary behaviours, such as walking and sitting [22, 24].

### Processing of raw accelerometer data

#### Detection of physical activity types and stationary behaviours

We used MATLAB software (Acti4) and information from the diaries to process the accelerometer raw data into *daily* time (i.e., duration in minutes per day) spent lying, sitting, standing, moving (i.e., small movements without regular walking while in a standing posture), walking, walking *slow* (<100 steps/min), walking *fast* (≥100 steps/min) [25], climbing stairs (i.e., both ascending and descending), cycling, running, and rowing as well as number of steps taken. Acti4 detects these physical behaviours through an algorithm based on thresholds of inclinations and standard deviations of accelerations that has been described in detail elsewhere [22].

#### Reference measurements

The daily reference measurements across the measurement period were identified by visual inspection of accelerometer inclinations at the time periods given in the diary. The reference measurement was used for detecting the angle between the axis of the accelerometer and the axis of the thigh that were used in Acti4’s activity-detection algorithm.

#### Validity of Acti4

With the exception of climbing stairs (sensitivity: 75.4%; specificity: 99.7%), the sensitivity has been found to be 90.4–99.4% and the specificity 93.1–100.0% across all activity types during standardised and semi-standardised conditions [22, 24].

#### Quality control, time in bed and non-wear time

By visual inspection of the activity classification over time, we investigated any abnormalities (e.g., only detected rowing or total lack of detected sitting).

We defined participants’ daily time spent in bed using a combination of diary (i.e., bedtime/get up time) and accelerometer data. Inconsistencies of more than 15 min between self-reported bedtime/get up-time and Acti4-detected lying/non-lying activity types (i.e., identified by visual inspection of the activity classification over time) were manually adjusted by setting the time to the closest five-minutes of the observed lying/non-lying activity.

Acti4 uses the following set of rules to detect non-wear time: Periods of <10 min without recorded movement were not regarded as non-wear time. Periods between 10 and 90 min were classified as non-wear time if 1) the vector sum of the standard deviation of acceleration was >0.5G for any second during a 5-s interval immediate before the period without recorded movement, and 2) the accelerometer was placed in a horizontal position (±5°). Periods >90 min were always considered as non-wear time [22]. In addition to the automatic detection of non-wear time by Acti4, non-wear time was also operator-defined by information given in the diary and through the visual inspection of the activity classification over time.

### Inclusion and exclusion criteria

To achieve reliable measurements of physical activity types and stationary behaviours, only individuals having *≥5 days* of measurements with *≥16 h* of recordings per 24-h day were included in the analyses, regardless of whether it was weekdays or weekend days. All days marked as ‘sick days’ in the diaries were excluded.

### Definition of variables

#### Physical activity types and stationary behaviours

As outcome variables, we used the individual daily mean time spent cycling, moving, walking (i.e., sum of all walking regardless of walking cadence), walking slow, walking fast, climbing stairs (up/down), running, standing, in sedentary behaviour (i.e., sum of lying and sitting), in light intensity physical activity (LIPA) (i.e., sum of moving and walking slow), in moderate-to-vigorous physical activity (MVPA) (i.e., sum of cycling, walking fast, climbing stairs, running and rowing), in bed, and number of steps taken per day (i.e., only accumulated during *waken* hours except from time in bed).

Additionally, based on available literature and theoretical considerations, we defined the following thresholds to reflect potentially health-related levels of physical activity types and stationary behaviours: cycling ≥15 min/day [3, 4], walking ≥1.5 h/day [26], walking fast (i.e., ≥100 steps/min) ≥30 min/day [16, 25], running ≥2.86 min/day reflecting 20 min/week [15], standing ≥4 h/day [27], ≥10 h/day of sedentary behaviour [8, 9], ≥2 h/day of LIPA [9], ≥30 min/day of MVPA [9] and < 5000 steps/day [28]. We calculated the frequency and prevalence of study participants spending time above or below the thresholds.

#### Variables for stratified analyses

We stratified our outcome variables by sex, age, level of education and body mass index (BMI). Age was categorised into the following age groups: 20 to <35, 35 to <50, 50 to <65, 65 to <75 and ≥75 years. The question regarding level of education, *“What education have you completed since you left municipal primary and lower secondary school?”* had the following response categories: *“No education”*; *“Short education (*≤*3 years with books)”*; *“Vocational or similar education (1-3 years)”*; *“Higher education (≥3 years, e.g., teacher, nurse or similar)”*; and *“University education”*. BMI was categorised into *underweight* (<18.5 kg/m^2^), *normal weight* (18.5-<25.0 kg/m^2^), *overweight* (25.0-<30.0 kg/m^2^) and *obese* (≥30 kg/m^2^) according to the WHO classification [29]. Because of a low number of underweight individuals (*n* = 15), we merged underweight with the normal weight category in the stratified analyses.

#### Variables for descriptive analyses

We used the following variables for descriptive purposes. Waist-hip ratio was calculated by dividing the waist circumference with the hip circumference. Mean systolic and diastolic blood pressure were calculated using the two last blood pressure measurements. If only one measurement was taken, this value was used as the participant’s mean. Blood pressure was categorised into *normal* (systolic: <140 mmHg and/or diastolic: <90 mmHg; i.e., including high normal), *grade 1 hypertension* (systolic: 140-≤159 mmHg and/or diastolic: 90-≤99 mmHg), *grade 2 hypertension* (systolic: 160-≤179 mmHg and/or diastolic: 100-≤109 mmHg), and *grade 3 hypertension* (systolic: ≥180 mmHg and/or diastolic: ≥110 mmHg) [30]. Smoking was assessed with the questions *“Do you smoke?”* and *“If no, have you previously smoked?”* with response categories *“Yes”* and *“No”*. We categorised study participants as smokers, previous smokers and non-smokers.

### Statistical analysis

Participant characteristics and outcome variables were described using medians with the first and third quartiles (Q1-Q3) and frequencies with percentages (%) as appropriate. We used medians instead of means because some of the continuous variables had skewed distributions.

To identify potential sources of selection bias, we compared the characteristics of the individuals that 1) did *not* give consent to wear accelerometers with those that gave consent, and 2) did *not* fulfil the inclusion criteria (i.e., ≥5 days with ≥16 h/day of accelerometer data) with those fulfilling the criteria, by assessing 95% confidence intervals (CIs) of medians and proportions.

Differences between population groups (i.e., sex, age groups, levels of education and BMI) for time spent in physical activity types, stationary behaviours, bed, and number of steps taken per day were assessed using Kruskal-Wallis rank sum tests (i.e., a *p*-value <0.05 were considered to indicate a difference between groups). We used 95% CIs to assess *which* groups that were different. Similarly, differences in the number of individuals spending time above or below the pre-specified health-related thresholds between population groups were assessed using Pearson’s Chi-squared test with Yate’s continuity correction and 95% CIs for proportions. The CIs for medians and proportions were calculated using the normal approximation method and the Wilson’s score method, respectively [31].

The distribution of both cycling and running time were skewed with a high number of individual means being equal or close to zero. Hence, to better illustrate the distribution of cycling and running time among those performing these activities, we presented the median time with Q1 and Q3 among those with an individual mean >0 min and >10 s, respectively. These time thresholds were chosen to exclude individuals not cycling and to exclude running time estimates that most likely were the result of misclassification.

#### Post hoc analyses

A combination of excessive sedentary behaviour and low time spent in MVPA is likely to increase the risk of mortality [11, 32]. Walking fast (i.e., ≥100 steps/min) corresponds to at least moderate intensity for most adults [25]. Based on this, we performed post hoc analyses to investigate how many participants and who (i.e., differences between population groups) that were both sedentary ≥10 h/day and did *not* walk fast ≥30 min/day and hence at potentially higher risk of premature mortality.

We used the statistical software R (version 3.5.1) for the analyses (https://www.r-project.org/).

## Results

### Final study population

**Fig. 1:**
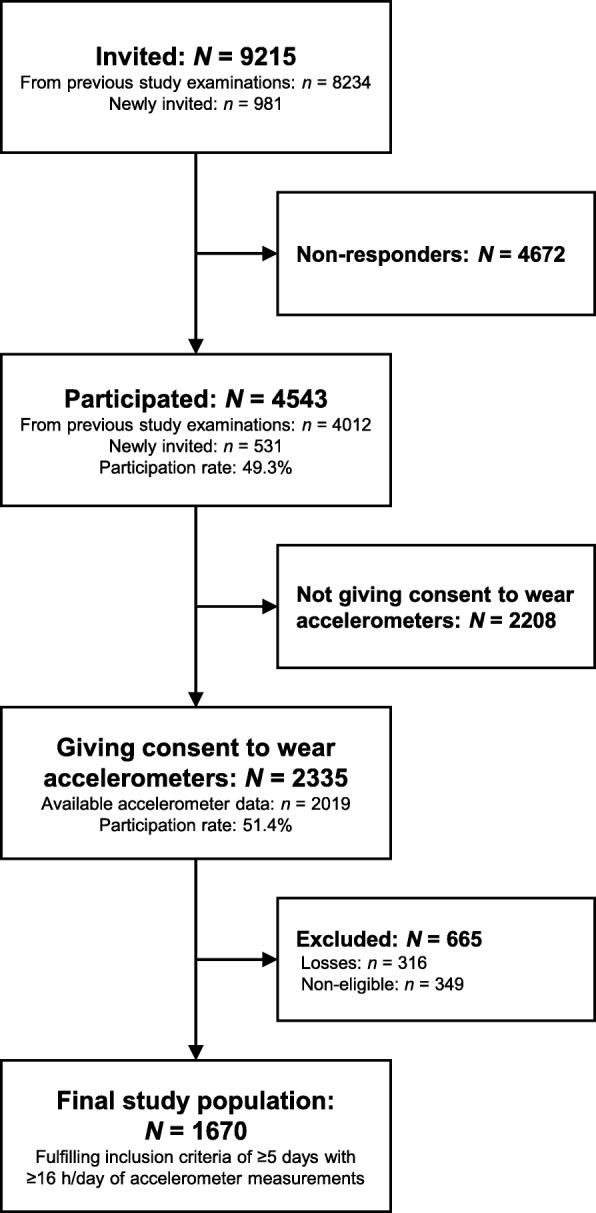
Formation of the final study population of participants with accelerometer-based measurements of physical activity and stationary behaviours in the fifth examination of the Copenhagen City Heart Study (Denmark). *N*/*n* is number of participants

In the *fifth* examination, 4543 individuals chose to participate out of 9215 invited (participation rate: 49.3%). Of these, 2335 gave consent to wear accelerometers (participation rate: 51.4%). After processing the raw accelerometer data, data from 2019 individuals were available of which 1670 fulfilled the inclusion criteria (82.7% of 2019) (Fig. 1).

The characteristics of the study population are presented in Table 1. The median wear time of accelerometers was 23.8 h/day and the median number of valid days was 6 days. The final study population consisted of 57.4% women. The median age was 61.8 years (Q1-Q3: 48.6–72.6). Similar proportions had a vocational education, higher education or a university education (25.4, 25.2 and 27.3%, respectively). The median BMI was 25.2 kg/m^2^. About 43% rated their general health as good. Finally, about 17% were smokers. Some variables had missing values; however, the proportion was for most variables <1%.

**Table 1:** Characteristics presented as frequencies with percentages or medians with first and third quartiles of 1670 Copenhageners (Denmark) participating in the fifth examination of the Copenhagen City Heart Study

Characteristics	*n* (%) / Median (Q1-Q3)
Accelerometer wear time	1670 (100.0)
Median h/day	23.8 (23.1–24.0)
Number of valid days of measurement	1670 (100.0)
Median number of days	6.0 (6.0–6.0)
Sex distribution	1670 (100.0)
Women	958 (57.4)
Men	712 (42.6)
Age	1670 (100.0)
Median years	61.8 (48.6–72.6)
Age group (years)	1670 (100.0)
20 - <35	196 (11.7)
35 - <50	250 (15.0)
50 - <65	522 (31.3)
65 - <75	431 (25.8)
≥75	271 (16.2)
BMI	1668 (99.9)
Median (kg/m^2^)	25.2 (22.8–28.0)
BMI, WHO classification	1668 (99.9)
Underweight	15 (0.9)
Normal	799 (47.9)
Overweight	620 (37.2)
Obese	234 (14.0)
WHR	1662 (99.6)
Median WHR	0.9 (0.8–0.9)
Level of education	1664 (99.6)
No [further] education	190 (11.4)
Short education (up to 3 years)	178 (10.7)
Vocational education (1–3 years)	423 (25.4)
Higher education (≥3 years)	419 (25.2)
University education	454 (27.3)
Longest type of occupation since completion of education	1664 (99.6)
Self-employed	158 (9.5)
Skilled/trained	341 (20.5)
Unskilled	161 (9.7)
“White-collar”/non-manual worker	863 (51.9)
Housewife/house husband	21 (1.3)
Student	86 (5.2)
Unemployed/retired	34 (2.0)
Household income (before tax)	1635 (97.9)
<100,000 DKK	60 (3.7)
100,000–200,000 DKK	304 (18.6)
200,000–400,000 DKK	469 (28.7)
400,000–600,000 DKK	287 (17.6)
600,000–800,000 DKK	221 (13.5)
>800,000 DKK	294 (18.0)
Civil status/marital status	1665 (99.7)
Married/cohabiting	1008 (60.5)
Unmarried	307 (18.4)
Separated/divorced	202 (12.1)
Widow/widower	148 (8.9)
Self-rated fitness compared to peers	1664 (99.6)
Same	793 (47.7)
Better	612 (36.8)
Worse	259 (15.6)
Smoking status	1639 (98.1)
Current smoker	286 (17.4)
Previous smoker	729 (44.5)
Non-smoker	624 (38.1)
Systolic blood pressure	1656 (99.2)
Median (mm Hg)	135.0 (122.5–150.5)
Blood pressure classification	1656 (99.2)
Normal	945 (57.1)
Grade 1 hypertension	567 (34.2)
Grade 2 hypertension	112 (6.8)
Grade 3 hypertension	32 (1.9)
Self-reported general health	1662 (99.5)
Excellent	139 (8.4)
Very good	532 (32.0)
Good	716 (43.1)
Less good	242 (14.6)
Poor	33 (2.0)

*n*, number of participants

*Q1-Q3,* first and third quartile

*BMI*, body mass index

*WHR*, waist-hip ratio

*DKK*, Danish kroner

Blood pressure classification is based on the 2013 European Society of Hypertension/European Society of Cardiology guidelines for the management of arterial hypertension. The normal category includes high normal

### Losses and exclusions

A higher proportion of participants *not* giving consent to wear accelerometers were aged ≥75 years, were classified as obese, had no education, had had a white-collar occupation or been housewife/house husband for the longest time since completion of education, were widow/widower, reported their fitness to be worse compared to their peers, and reported their general health to be *less good* or *poor*, compared to consenting participants. With regards to leisure time physical activity, a higher proportion of non-consenting individuals reported being sedentary, while a lower proportion reported “regular physical activity and exercise (moderate physical activity)” compared to consenting participants. For details see Table A2.1 in Additional file 2.

The participants that did *not* fulfil the inclusion criteria (i.e., ≥5 days with ≥16 h/day) were younger, and a higher proportion had a university education, were students, unmarried, non-smokers, and had a lower systolic blood pressure, while a lower proportion were previous smokers, compared to eligible participants. Furthermore, a higher proportion reported “mainly sedentary work”, while a lower proportion reported “sitting or standing, from time to time walking during work”. Finally, a higher proportion reported “more strenuous [leisure time] physical activity” compared to eligible participants. See Table A2.2 in Additional file 2 for details.

Accelerometer data from 316 participants were lost due to different reasons. The primary reasons were incorrect initialisation of the accelerometers (i.e., non-recordings), accelerometers lost in postal services, and <1 h of wear time (i.e., data was not processed if total wear time <1 h).

### Time spent in physical activity types and stationary behaviours

#### Overall population

The results for the overall population are presented in Table 2 and Table A4.1 in Additional file 4. Among the 61% of the study population that cycled during the measurement period, the median time spent cycling was 8.3 (Q1-Q3: 2.5–18.2) min/day. Overall, about 20% cycled on average ≥15 min/day and 27% cycled ≥10 min/day.

**Table 2:** Time spent in physical activity types, stationary behaviours and number of steps/day among 1670 adult Copenhageners (Denmark) participating in the fifth examination of the Copenhagen City Heart Study

Behaviour, min/day	Overall population *N* = 1670 Median (Q1-Q3)
Time in bed	479.50 (445.00–519.17)
Sedentary behaviour	579.07 (508.86–645.79)
Standing	182.51 (143.22–225.51)
Moving	68.66 (53.25–88.11)
Walking	82.58 (63.32–106.32)
Walking slow	19.48 (13.34–27.95)
Walking fast	55.07 (40.29–73.84)
Climbing stairs (up/down)	4.37 (2.37–6.80)
Number of steps/day	9288.08 (6932.22–12,003.23)
LIPA	91.29 (70.6–113.93)
Cycling	1.24 (0.00–11.51)
Cycling, among those cycling >0 min/day Prevalence	8.31 (2.48–18.20) 60.84%
Running	0.12 (0.04–0.46)
Running, among those running >10 s/day Prevalence	0.61 (0.27–4.00) 43.77%
MVPA	70.72 (50.36–93.52)

All estimates are in min/day except for number of steps/day that is presented in number of steps taken per day

*N*, number of observations

*Q1-Q3*, first and third quartile

Moving consists of small movements without regular walking during a standing posture

Walk slow and fast corresponds to walking <100 and ≥100 steps/min, respectively

Prevalence refers to the prevalence of cycling >0 min/day and running >10 s/day (on average), respectively

*s*, seconds

*LIPA*, light intensity physical activity

*MVPA*, moderate-to-vigorous physical activity

The median time spent walking, walking fast and climbing stairs (up/down) was 82.6 (Q1-Q3: 63.3–106.3), 55.1 (40.3–73.8) and 4.4 (2.4–6.8) min/day, respectively. About 42% walked on average ≥1.5 h/day and 87.5% walked fast ≥30 min/day.

Among the 44% that on average ran >10 s/day during the measurement period, the median time spent running was 0.6 (Q1-Q3: 0.3–4.0) min/day. We found that 13.2% ran, on average, what corresponds to ≥20 min/week.

The median time spent standing was 182.5 (Q1-Q3: 143.2–225.5) min/day and 19.2% spent on average ≥4 h/day standing. We found that the median time spent in sedentary behaviour was 579.1 (Q1-Q3: 508.9–645.8) min/day, and that 40.7% of the study population spent on average ≥10 h/day being sedentary.

The median time in LIPA and MVPA was 91.3 (Q1-Q3: 70.6–113.9) and 70.7 (50.4–93.5) min/day, respectively. The proportions spending on average ≥1.5 h/day in LIPA and ≥30 min/day in MVPA was 19.3 and 91.8%, respectively.

The median number of steps/day was 9288.1 (Q1-Q3: 6932.2–12,003.2). The proportion taking on average <5000 steps/day was 9.6%. Finally, the median time in bed was 479.5 (Q1-Q3: 445.0–519.2) min/day.

#### Stratified analyses

**Fig. 2:**
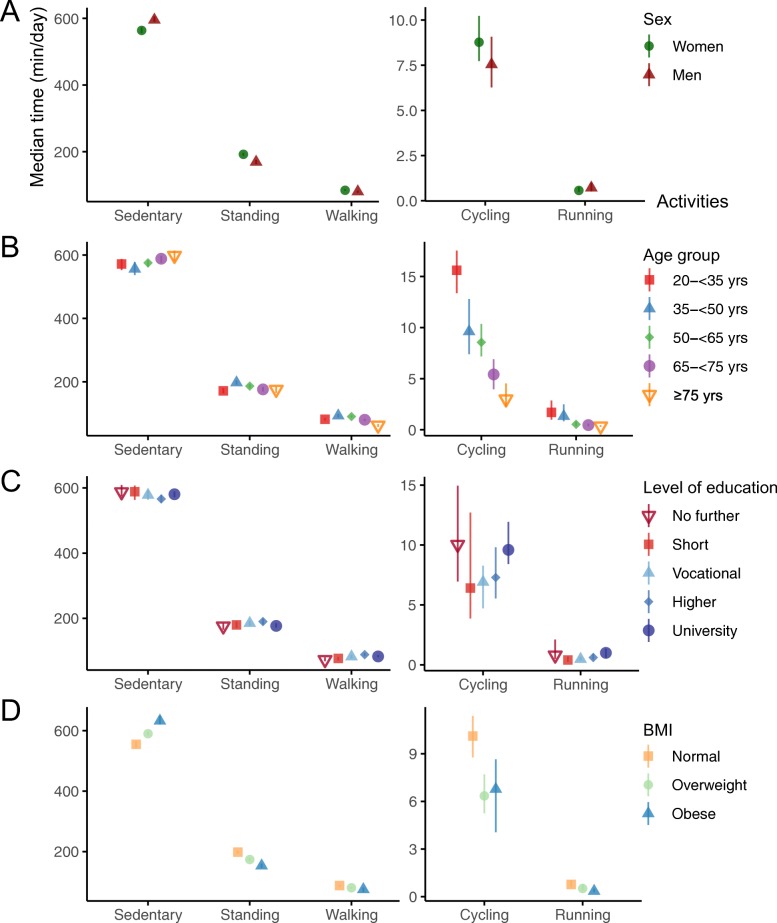
Median time spent sedentary, standing, walking, cycling and running among 1670 Copenhageners (Denmark) participating in the fifth examination of the Copenhagen City Heart Study, stratified by **a** sex, **b** age, **c** level of education and **d** BMI. Vertical lines indicate 95% confidence intervals. The estimated cycling and running times are based only on participants cycling and running during the measurement period, defined as an individual mean >0 min/day and >10 s/day, respectively. BMI is body mass index. The normal weight category includes 15 participants being underweight

Women spent more time walking, walking fast, standing, in MVPA, and in bed, and took a higher number of steps/day compared to men, who on the other hand had a longer duration of time spent sedentary and walking slow. When we stratified the prevalence of spending time above or below the specified health-related thresholds by sex, similar differences were found. For details, see Fig. 2a, and Table A3.1 and Table A4.1 in Additional files 3 and 4, respectively.

The older age groups spent in general less time cycling, walking, walking fast, climbing stairs (up/down), running, standing, in MVPA, and took fewer number of steps/day compared to the younger age groups, while the opposite was observed for sedentary behaviour. For walking slow and time in LIPA, an *inverse* U-shaped distribution peaking in the 50 to 64 and 65 to 74 years age groups was observed. Conversely, a U-shaped distribution with the shortest duration in the 35 to 50 years age group was observed for time in bed. Similar observations were found for the age-stratified prevalence of spending time above or below specified health-related thresholds (Fig. 2b, and Table A3.2, and Table A4.2 in Additional files 3 and 4, respectively).

Individuals with higher educational levels spent in general more time cycling (i.e., overall), climbing stairs (up/down), running, and in MVPA compared to individuals with lower educational levels. Among those cycling, we found no differences in cycling time; however it should be emphasised that the CIs were wide due to low number of participants in some of the educational groups. An inverse U-shaped distribution across educational levels was seen in the following activities: walking, walking fast, standing and number of steps/day, with the longest durations found in the *higher education* group; and moving, walking slow, and LIPA with the longest durations found in the *vocational education* group. Slightly longer durations of sedentary behaviour were observed among those *without further education*, *short education* and *university education* compared to the other groups. No clear differences were seen for time in bed. Overall, we found similar observations when the prevalence of spending time above or below specified health-related thresholds was stratified by level of education. See Fig. 2c, and Table A3.3 and Table A4.3 in Additional files 3 and 4, respectively for details.

We found longer durations of sedentary time among overweight and obese individuals compared to individuals of normal weight, and the opposite for time spent cycling, moving, walking (i.e., overall, slow, fast, and climbing stairs), running, standing, in LIPA and MVPA, and number of steps/day. Similar differences were found when the prevalence of spending time above or below specified health-related thresholds were stratified by BMI. For details, see Fig. 2d, and Table A3.4 and Table A4.4 in Additional files 3 and 4, respectively.

#### Post hoc analyses

Our post hoc analyses showed that 8.9% of all participants were sedentary ≥10 h/day and walked fast < 30 min/day (and that 31.9% were sedentary ≥10 h/day and walked fast ≥30 min/day). When stratified, we found a higher proportion among older individuals than younger, among those without a further education or with a vocational education compared to those with a higher or a university education, and a higher proportion among overweight and obese individuals compared to those with normal weight (see Table A4.1-A4.4 in Additional files 4 for details).

## Discussion

### Summary of findings

To our knowledge, this is the first study reporting accelerometer-based estimates about time spent cycling, walking, running, standing and sedentary in an adult general population. We found a long duration and high prevalence of cycling and walking among the study participants. However, many Copenhageners also spent a lot of time being sedentary.

A shorter duration of cycling, walking and running was found among older individuals, individuals with the lowest educational levels and individuals being overweight and obese. The longest duration of time spent sedentary was found among men, and individuals being older, overweight and obese, but no differences were seen between educational levels.

### Interpretation of findings

Considering the high proportion of older individuals in our study population (i.e., 42% ≥65 years), we believe the daily cycling time and the prevalence of cycling (i.e., 8.31 min/day among the 61% cycling and about 20% cycled ≥15 min/day) is relatively high, and reflects the strong cycling culture in Copenhagen, the *“City of Cyclists”* [12]. Cycling is well-documented to lower the risk of mortality [3, 4, 6, 17, 26], cardiovascular disease and type 2 diabetes [5, 7], and is associated with other health outcomes, such as lower BMI [33], and higher health-related quality of life among elderly [29]. The observed cycling is hence likely to have a considerable positive effect on the public health of residents of Copenhagen [14]. Looking beyond health, cycling has both economic [34] and environmental benefits [35].

To our knowledge, this is the first study reporting cycling time in a general population measured with accelerometers during a week. Consequently, comparison of our results with other studies is limited. However, the median cycling time of 8.31 min/day (58.17 min/week) among those cycling is slightly *shorter* compared to self-reported estimates found in other cohort studies, ranging from 25.7 min/day in Denmark [4] to 10.6 min/day in the Netherlands [36]. In contrast, the prevalence of cycling in our study (61%) is *higher or similar* compared to estimates based on self-reported data reported in other studies of Belgian, Danish and Dutch general populations, ranging from 43% [37] to 69% [6, 17, 37]. These differences may be explained by the poor agreement between self-reported and direct measurements of physical activity [18], and other factors such as differences in attributes of the built environment known to affect cycling levels [37].

We found that 88% of the study population walked fast (i.e., ≥100 steps/min) ≥30 min/day. For most individuals, a walking cadence of 100 steps/min corresponds to physical activity of moderate intensity [25]. Thus, almost 90% of our study population fulfil a large part of WHO’s physical activity recommendations of ≥150 min of moderate-intensity physical activity per week by walking only [38]. However, comparison of these numbers with recent global and regional estimates of insufficient physical activity [39] is challenging since our estimates include all walking during waken hours, and the WHO recommendations are based on the accumulation of MVPA in bouts of ≥10 min [38]. Future comparison may be easier, since the Physical Activity Guidelines for Americans now have dropped the bout-requirement [40]. Walking with moderate or higher intensity is known to lower the risk of premature mortality [26, 41] and has beneficial effects on cardiovascular disease risk factors [2]. Thus, our finding of a high walking time of at least moderate intensity highlights the potential of improving public health through the promotion of walking [42, 43].

Similar to cycling, there are few previous studies of the general population with comparable accelerometer-based measurements of walking. The median walking time (i.e., 82.6 min/day) of our study is *longer* than self-reported [41] and accelerometer-based (i.e., count-based) [44] walking estimates from general population studies from high- and upper-middle-income countries. Again, self-reported measurements have in general low agreement with direct measurement of physical activity [18]. However, the count-based estimates of walking time in the NHANES 2005–2006 are substantially shorter (i.e., sum of slow, moderate and brisk walking: 28 min/day) [44]. Even if the categories “purposeful steps” (i.e., 40–50 steps/min; 66.9 min/day) and “faster locomotion” (i.e., ≥120 steps/min; 1.5 min/day) is added, the estimated walking time is still considerably shorter [44]. Interestingly, the number of steps/day in the NHANES 2005–2006 (i.e., uncensored: 9685 steps/day) [44] is similar to our findings of 9288 steps/day. Some of the differences in walking time are hence likely explained by differences in the accelerometer position and processing of the data.

We found that 41% spent ≥10 h/day sedentary. Several studies indicate that a sedentary time of 10–11 h/day or more increase the risk of incident cardiovascular disease, type 2 diabetes and mortality [8–10]. Thus, a relatively high proportion of the adult population of Copenhagen may be at increased risk of cardiometabolic disease and mortality. This may in particular concern those with concurrent low levels of fast walking (i.e., older individuals, those with lower educational levels, and overweight or obese individuals), since the detrimental effects from excessive sedentary behaviour are dependent on the level of MVPA [11, 32]. Hence, this risk should be seen in light of the previously discussed relatively high levels of cycling and walking fast, which may reduce the risks associated with excessive sedentary behaviour [11, 32].

Our median sedentary time of 579 min/day is *similar* [20, 44, 45] *or shorter* [46] than other accelerometer-based estimates from cohort studies of general populations from high-income countries. Importantly, these studies used count-based classification of sedentary time (e.g., <100 cpm), which may not be directly comparable to our results that are based on posture-detected estimation of sitting and lying. For example, lying, sitting, and standing are all likely to result in <100 cpm. Hence, the differentiation of standing from sitting and lying may partly explain why our estimates are slightly lower than those reported by Diaz et al. that defined sedentary behaviour as 0–49 cpm [46]. Furthermore, the prevalence of being sedentary ≥10 h/day (i.e., 41%) is *higher* compared to other accelerometer-based studies of the general adult population (e.g., 23–24%) [9, 20]. This can be a result of the relatively high proportion of elderly (i.e., who have a longer duration of sedentary behaviour) in our study population compared to other studies.

We found a shorter duration of cycling, walking and running among older individuals, individuals with the lowest educational levels and individuals with a higher BMI. Although reported in terms of *overall physical activity*, this is in agreement with previous studies [47–49]. We also found that women spent more time in some physical activity types (i.e., longer duration of walking, walking fast, MVPA, and a higher number of steps/day), which is contrary to what has been reported in summaries of previous studies where male sex is associated with higher levels of physical activity [48, 49]. With regards to sedentary behaviour, we found a longer duration among men, individuals being older, and individuals being overweight and obese. These findings is in line with previous research [50, 51]. However, we did not find any differences between educational levels, which has been found in previous studies [50, 51].

### Methodological considerations

Considering the participation rate in the fifth examination of the CCHS (49.4%) and the percentage wearing accelerometers (51.4%), the risk of selection bias should be acknowledged. Based on differences in self-reported leisure time physical activity between participants not giving and giving consent to wear accelerometers (i.e., those not giving consent reported more sedentary behaviour and less regular physical activity and exercise), it is possible that our group-level estimates of sedentary behaviour and more vigorous activity types (e.g., cycling, walking fast and running) are under- and overestimated, respectively.

The high validity of the Acti4 software in detecting physical activity types and body postures from thigh-worn accelerometers [22, 24] is a strength of our study. Detection of cycling by Acti4 is based on continuous pedalling. This means that interrupted pedalling for >15 s during a cycling trip (e.g., waiting at traffic lights or freewheeling) will be recorded as time spent sitting or standing depending on the position of the right thigh, and not cycling. This can explain some of the previously mentioned differences between self-reported cycling time and our cycling time estimates, since self-reported estimates most likely include the recalled total travel time (e.g., going “from A to B”).

We chose our inclusion criteria (i.e., ≥5 days with ≥16 h/day) to achieve estimates of physical activity and stationary behaviours with high reliability. This excluded 349 (17.3%) participants from the analyses that were slightly different, again leading to further selection of the study population. We acknowledge the trade-off between external validity of the results and reliability of the measurements. However, no differences in time spent in the activities were seen when we compared our findings with those based on a less conservative inclusion criteria (i.e., ≥1 day with ≥16 h/day); therefore, this should not have a significant impact on our findings.

Our results are based on the individual mean time spent in the physical behaviours across the measurement period. Given our inclusion criteria (≥5 days of measurements with ≥16 h of recordings per 24-h day), the measurement period would for most individuals include both weekdays and weekend days. We acknowledge that physical activity patterns may be different on weekdays and weekends, but believe that the investigation of this lies beyond the scope of this paper.

We do not have information about where the measured physical behaviours take place (i.e., geographical location). However, we believe that reaching risk groups can be achieved despite the lack of information about where they are physically active. With the information about how people are active, city planners could, for example, nudge the target group (and potentially all of us!) towards a more active lifestyle (e.g., active transportation by cycling or walking, climbing stairs instead of using escalators or elevators, etc.). Policy makers could support local grassroots initiatives (e.g., running communities) or sports clubs. Researchers and others could mobilise knowledge about easy ways to increase physical activity to the target groups (e.g., through interest groups, senior- or patient organisations). Finally, different social media channels may offer other possibilities to reach risk groups in society.

### Perspectives

In the light of substantial evidence of health benefits from cycling and walking, the long duration and high prevalence of cycling and walking found in this study population may contribute to a substantial reduction in the risk of developing NCDs and mortality [2, 3, 5, 7, 16, 26, 41].

Our findings may reflect Copenhagen’s strategy and investments over the past two decades to increase active transportation [12]. We believe city planning has a great potential in creating active, healthy societies that facilitate physical activity as part of daily living, promotes health and prevents NCDs [1, 52]. This should be a high priority for policy makers globally. However, our results also show that many Copenhageners spend much time sedentary, and that individuals being older, those with a short education and individuals being overweight and obese are least active through cycling, walking and running. WHO's Global action plan on physical activity 2018–2030 has the vision of *“more active people for a healthier world”* (1)*.* The data in the present study are highly relevant for stakeholders to tailor initiatives at different societal levels to promote physical activity among the least active residents of Copenhagen. As previously discussed, this requires both population-level and individually focused approaches entailing collaboration between different sectors, such as policymaking, public health, city planning, business and industry, education, health care, mass media, and others [1, 40, 42].

Finally, these data provide unique opportunities to gain new knowledge about the role of physical activity types and stationary behaviours in both the development and prevention of NCDs. For example, by linkage of these estimates with national register data and by testing associations with risk factors for NCDs.

## Conclusions

We found a long duration and high prevalence of cycling and walking, but also that many adult Copenhageners spent much time sedentary. Population groups with low participation in physical activities such as cycling and walking (e.g., older individuals, individuals with a low level of education, and individuals being overweight and obese) should be targeted in future initiatives towards an active, healthy society. Encouraging the least active to be more active should be of high priority to prevent and lower the burden from NCDs. We hope that this study about time spent in specific physical activity types and stationary behaviours in the population of Copenhagen can form a benchmark for policy makers, city planners and researchers globally.

## Supplementary information

**Additional file 1.** Overview of questions. Table providing an overview of the questions used for the purpose of this study.

**Additional file 2.** Investigation of potential selection bias. Tables with comparison of the characteristics of the individuals that did and did not give consent to wear accelerometers, and of the individuals that did and did not fulfil the inclusion criteria.

**Additional file 3.** Time spent in physical activity types, stationary behaviours and number of steps/day. Tables presenting the time spent in physical activity types, stationary behaviours and number of steps taken per day stratified by sex, age, level of education and BMI.

**Additional file 4.** Prevalence of spending time above/below health-related thresholds in physical behaviours. Tables presenting the prevalence of spending time above or below health-related thresholds in physical activity types and stationary behaviours, overall and stratified by sex, age, level of education and BMI.

## Data Availability

The data generated and analysed during the current study are not publicly available; however, anybody can apply for the use of data by contacting the steering committee [53].

## Abbreviations

BMI: Body mass index
CCHS: Copenhagen City Heart Study
CI: Confidence interval
LIPA: Light intensity physical activity
MVPA: Moderate-to-vigorous physical activity
NCD: Non-communicable disease
Q1: First quartile
Q3: Third quartile
WHO: World Health Organization
