# Supplementary material for: Time spent cycling, walking, running, standing and sedentary: a cross-sectional analysis of accelerometer-data from 1670 adults in the Copenhagen City Heart Study: Physical behaviours among 1670 Copenhageners
Source: BMC Public Health. 2019 Oct 24;19:1370. doi: 10.1186/s12889-019-7679-z (PMC6814105; doi:10.1186/s12889-019-7679-z)
Supplement: Supplementary file 1 — Additional file 1. Overview of questions. Table providing an overview of the questions used for the purpose of this study. [file 12889_2019_7679_MOESM1_ESM.pdf]

Additional file 1

**Table A1.1.** Overview of questions and responses from the questionnaire in the fifth examination of the Copenhagen City Heart Study (Denmark) used in the present study	
**Question**	**Possible responses**
Level of education	
What education have you completed since you left municipal primary and lower secondary school?	No education<br>Short education (≤3 years with books)<br>Vocational or similar education (1-3 years)<br>Higher education (≥3 years, e.g., teacher, nurse or similar)<br>University education
Occupation/employment	
What occupation have you had for the longest time, since you completed your education/school years?	Self-employed<br>Skilled/trained<br>Unskilled<br>White-collar/non-manual worker<br>Housewife/ house husband<br>Student<br>Unemployed/retired
Household income	
What was your total household income before tax last year?	<100 000 DKK<br>100 000 – 200 000 DKK<br>200 000 – 400 000 DKK<br>400 000 – 600 000 DKK<br>600 000 – 800 000 DKK<br>>800 000 DKK
Civil status	
At the present moment, are you?	Married/cohabiting<br>Unmarried<br>Separated/divorced<br>Widow/widower
Occupational physical activity	
State your physical activity level during work within the last year (also completed by housewives, students, and currently unemployed, while retirees without actual employment are asked to go to next question)	I. Mainly sedentary work (e.g., paperwork/desk work; housewife/house husband without underage children but with domestic help)<br>II. Sitting or standing, from time to time walking (e.g., shop assistant, teacher; housewife/house husband, who wash and clean, without underage children)<br>III. Walking, from time to time lifting (e.g., postman; nursing staff; housewife/house husband, who wash and clean, with one or more underage children)<br>IV. Heavy physical work/manual work (e.g., remover, concrete worker)

**Table A1.** (Continued)	
Leisure time physical activity	
State your physical activity level during in your leisure time (including transportation to and from work) within the last year	I. Being almost completely inactive or active in light physical activity for less than 2 h/week (e.g., reading, watching television, cinema)<br>II. Light physical activity for 2-4 h/week (e.g., walks, cycling trips, light gardening, light calisthenics)<br>III. Light physical activity for >4 h/week or more strenuous physical activity 2-4 h/week (e.g., brisk walking and/or fast cycling, heavy gardening, heavy calisthenics, where you sweat or get out of breath)<br>IV. More strenuous physical activity for >4 h/week or regular hard physical exercise and possibly competitions several times a week
Self-rated fitness compared to peers	
How do you rate your fitness compared to your peers?	Same<br>Better<br>Worse
Smoking status	
Do you smoke?<br>If no, have you previously smoked?	Yes<br>No
Self-reported general health	
How do you think your health is all in all?	Excellent<br>Very good<br>Good<br>Less good<br>Poor
All questions have been freely translated from Danish to English for the purpose of this overview only.<br>DKK, Danish kroner	
