# Supplementary material for: Time spent cycling, walking, running, standing and sedentary: a cross-sectional analysis of accelerometer-data from 1670 adults in the Copenhagen City Heart Study: Physical behaviours among 1670 Copenhageners
Source: BMC Public Health. 2019 Oct 24;19:1370. doi: 10.1186/s12889-019-7679-z (PMC6814105; doi:10.1186/s12889-019-7679-z)
Supplement: Supplementary file 2 — Additional file 2. Investigation of potential selection bias. Tables with comparison of the characteristics of the individuals that did and did not give consent to wear accelerometers, and of the individuals that did and did not fulfil the inclusion criteria. [file 12889_2019_7679_MOESM2_ESM.pdf]

Additional file 2

Table A2.1. Comparison of characteristics of participants consenting and not consenting to wear accelerometers in the fifth examination of the Copenhagen City Heart Study (Denmark)

**Fifth examination of the Copenhagen City Heart Study** ***N*=4543 (100.0%)**	**Consenting** ***n*=2335 (51.4%)**	**Non-consenting** ***n*=2208 (48.6%)**
**Characteristics**	***n* (%) [95% CI] / Median [95% CI]**	***n* (%) [95% CI] / Median [95% CI]**
Sex distribution	2335 (100.0)	2208 (100.0)
Women	1330 (56.96) [54.92-58.98]	1,233 (55.84) [53.74-57.92]
Men	1005 (43.04) [41.02-45.08]	975 (44.16) [42.08-46.26]
Age	2335 (100.0)	2208 (100.0)
Median years	56.12 [55.30-57.07]	57.34 [56.40-58.83]
Age group (years)	2335 (100.0)	2208 (100.0)
20-<35	463 (19.83) [18.24-21.52]	403 (18.25) [16.67-19.94]
35-<50	383 (16.40) [14.94-17.98]	369 (16.71) [15.19-18.35]
50-<65	787 (33.70) [31.79-35.67]	672 (30.43) [28.53-32.41]
65-<75	512 (21.93) [20.28-23.67]	457 (20.70) [19.04-22.46]
≥75	190 (8.14) [7.08-9.34]*	307 (13.90) [12.50-15.43]*
BMI	2332 (99.87)	2116 (95.83)
Median (kg/m^2)	25.12 [24.94-25.29]	25.14 [24.97-25.40]
BMI, WHO classification	2332 (99.87)	2116 (95.83)
Underweight	24 (1.03) [0.68-1.55]	37 (1.75) [1.25-2.43]
Normal	1135 (48.67) [46.62-50.72]	1020 (48.20) [46.06-50.36]
Overweight	860 (36.88) [34.92-38.88]	724 (34.22) [32.20-36.29]
Obese	337 (14.45) [13.06-15.96]*	372 (17.58) [15.99-19.29]*
WHR	2325 (99.57)	2109 (95.52)
Median WHR	0.876 [0.870-0.880]	0.88 [0.876-0.886]
Level of education	2327 (99.66)	2191 (99.23)
No [further] education	269 (11.56) [10.30-12.95]*	321 (14.65) [13.21-16.22]*
Short education (up to 3 years)	228 (9.80) [8.64-11.10]	230 (10.50) [9.26-11.87]
Vocational or comparable education (1-3 years)	567 (24.37) [22.64-26.17]	532 (24.28) [22.51-26.14]
Higher education (≥3 years)	580 (24.92) [23.19-26.74]*	467 (21.31) [19.63-23.10]*
University education	683 (29.35) [27.52-31.26]	641 (29.26) [27.37-31.22]
Longest type of occupation since completion of education	2325 (99.57)	2188 (99.09)
Self-employed	214 (9.20) [8.08-10.47]	244 (11.15) [9.88-12.56]
Skilled/trained	467 (20.09) [18.49-21.79]	417 (19.06) [17.45-20.78]
Unskilled	226 (9.72) [8.56-11.01]	251 (11.47) [10.18-12.90]
"White-collar"/non-manual worker	1194 (51.35) [49.30-53.40]*	1031 (47.12) [45.01-49.24]*
Housewife/house husband	24 (1.03) [0.68-1.56]*	54 (2.47) [1.88-3.23]*
Student	148 (6.37) [5.42-7.45]	127 (5.80) [4.88-6.89]
Unemployed/retired	52 (2.24) [1.69-2.95]	64 (2.93) [2.28-3.74]
Household income (before tax)	2284 (97.82)	2123 (96.15)
<100,000 DKK	92 (4.03) [3.28-4.94]	115 (5.42) [4.51-6.49]
100,000 – 200,000 DKK	423 (18.52) [16.96-20.19]	454 (21.38) [19.67-23.20]
200,000 – 400,000 DKK	614 (26.88) [25.08-28.76]	588 (27.70) [25.81-29.66]
400,000 – 600,000 DKK	406 (17.78) [16.24-19.42]	337 (15.87) [14.36-17.51]
600,000 – 800,000 DKK	318 (13.92) [12.54-15.43]	244 (11.49) [10.18-12.94]
>800,000 DKK	431 (18.87) [17.30-20.55]	385 (18.13) [16.53-19.86]
Current civil status/marital status	2325 (99.57)	2185 (98.96)
Married/cohabiting	1402 (60.30) [58.28-62.29]*	1224 (56.02) [53.91-58.11]*
Unmarried	464 (19.96) [18.36-21.65]	448 (20.50) [18.84-22.27]
Separated/divorced	263 (11.31) [10.07-12.69]	239 (10.94) [9.68-12.34]
Widow/widower	196 (8.43) [7.35-9.65]*	274 (12.54) [11.20-14.02]*
Occupational physical activity	1563 (66.94)	1362 (61.68)
Mainly sedentary work	680 (43.51) [41.04-46.01]	643 (47.21) [44.53-49.90]
Sitting or standing, from time to time walking	549 (35.12) [32.77-37.56]	461 (33.85) [31.35-36.44]
Walking, from time to time lifting	296 (18.94) [17.04-20.99]	232 (17.03) [15.10-19.16]
Heavy physical work/manual work	38 (2.43) [1.75-3.36]	26 (1.91 [1.28-2.83]
Leisure time physical activity	2312 (99.01)	2152 (97.46)
Being almost completely inactive or active in light physical activity for <2 h/week	138 (5.97) [5.05-7.03]*	247 (11.48) [10.18-12.92]*
Light physical activity for 2-4 h/week	909 (39.32) [37.32-41.35]	856 (39.78) [37.71-41.88]
Light physical activity for >4 h/week or more strenuous physical activity 2-4 h/week	1089 (47.10) [45.05-49.16]*	880 (40.89) [38.81-43.01]*
More strenuous physical activity for >4 h/week or regular hard physical exercise and possibly competitions several times a week	176 (7.61) [6.58-8.79]	169 (7.85) [6.77-9.09]

Table A2.1. (Continued)

Self-rated fitness compared to peers	2323 (99.49)	2176 (98.55)
Same	1104 (47.52) [45.48-49.58]	993 (45.63) [43.53-47.76]
Better	864 (37.19) [35.23-39.20]	730 (33.55) [31.57-35.58]
Worse	355 (15.28) [13.86-16.82]*	453 (20.82) [19.14-22.60]*
Smoking status	2292 (98.16)	2152 (97.46)
Current smoker	415 (18.11) [16.56-19.76]	431 (20.03) [18.37-21.80]
Previous smoker	971 (42.36) [40.33-44.42]	897 (41.68) [39.59-43.80]
Non-smoker	906 (39.53) [37.52-41.57]	824 (38.29) [36.24-40.39]
Systolic blood pressure	2314 (99.10)	2090 (94.66)
Median (mmHg)	134.00 [133.00-135.00]	135.00 [134.0-136.5]
Blood pressure classification	2314 (99.10)	2090 (94.66)
Normal	1361 (58.82) [56.78-60.83]	1165 (55.74) [53.58-57.88]
Grade 1 hypertension	760 (32.84) [30.94-34.81]	685 (32.78) [30.77-34.84]
Grade 2 hypertension	152 (6.57) [5.61-7.67]	186 (8.90) [7.73-10.22]
Grade 3 hypertension	41 (1.77) [1.29-2.42]	54 (2.58) [1.96-3.38]
Self-reported general health	2322 (99.44)	2182 (98.82)
Excellent	211 (9.09) [7.96-10.35]	206 (9.44) [8.26-10.76]
Very good	751 (32.34) [30.45-34.30]	624 (28.60) [26.72-30.55]
Good	992 (42.72) [40.70-44.77]	886 (40.60) [38.54-42.70]
Less good	318 (13.70) [12.34-15.18]*	384 (17.60) [16.04-19.28]*
Poor	50 (2.15) [1.62-2.85]*	82 (3.76) [3.02-4.67]*

The non-consenting group includes 191 individuals with missing information on whether they agreed to wear accelerometers or not.

N/n, number of observations

CI, confidence interval, CIs were calculated using the Wilson score method for proportions and the normal approximation method for medians.

* indicate non-overlapping CIs between the groups.

BMI, body mass index

WHR, waist-hip-ratio

DKK, Danish kroner

Blood pressure classification is based on the 2013 European Society of Hypertension/European Society of Cardiology guidelines for the management of arterial hypertension. The normal category includes high normal.

Table A2.2. Comparison of characteristics of participants in the fifth examination of the Copenhagen City Heart Study (Denmark) fulfilling and not fulfilling inclusion criteria (≥5 days with ≥16h/day) with regards to the length of accelerometer measurements

**Participants with available accelerometer data** ***N*=2032 (100.0%)**	**Eligible** ***n*=1670 (82.2%)**	**Non-eligible** ***n*=362 (17.8%)**
**Characteristic**	***n* (%) [95% CI] / Median [95% CI]**	***n* (%) [95% CI] / Median [95% CI]**
Sex distribution	1670 (100.0)	362 (100.0)
Women	958 (57.37) [54.95-59.75]	215 (59.39) [54.12-64.46]
Men	712 (42.60) [40.25-45.05]	147 (40.61) [35.54-45.88]
Age	1670 (100.0)	362 (100.0)
Median years	58.05 [57.21-59.02]*	46.34 [41.33-49.82]*
Age group (years)	1670 (100.0)	362 (100.0)
20-<35	261 (15.63) [13.94-17.48]*	134 (37.01) [32.07-42.24]*
35-<50	265 (15.90) [14.17-17.73]	68 (18.78) [14.97-23.28]
50-<65	584 (34.97) [32.69-37.32]*	98 (27.07) [22.62-32.01]*
65-<75	403 (24.13) [22.11-26.27]*	45 (12.43) [9.30-16.38]*
≥75	157 (9.40) [8.07-10.93]*	17 (4.7) [2.85-7.56]*
BMI	1668 (99.88)	361 (99.72)
Median (kg/m^2)	25.16 [24.87-25.39]	25.02 [24.35-25.41]
BMI, WHO classification	1668 (99.88)	361 (99.72)
Underweight	15 (0.90) [0.52-1.52]	5 (1.39) [0.51-3.39]
Normal	811 (48.80) [46.38-51.23]	180 (49.86) [44.60-55.13]
Overweight	620 (37.17) [34.85-39.55]	136 (37.67) [32.70-42.92]
Obese	234 (14.0) [12.42-15.81]	45 (12.47) [9.33-16.43]
WHR	1664 (99.64)	360 (99.45)
Median WHR	0.878 [0.872-0.883]	0.852 [0.843-0.875]
Level of education	1664 (99.64)	362 (100.0)
No [further] education	190 (11.41) [9.95-13.07]	34 (9.39) [6.68-12.99]
Short education (up to 3 years)	178 (10.70) [9.27-12.31]	24 (6.63) [4.38-9.84]
Vocational or comparable education (1-3 years)	423 (25.42) [23.36-27.60]	80 (22.1) [18.00-26.80]
Higher education (≥3 years)	419 (25.18) [23.12-27.35]	97 (26.80) [22.36-31.73]
University education	454 (27.28) [25.17-29.50]*	127 (35.08) [30.21-40.27]*
Longest type of occupation since completion of education	1664 (99.64)	361 (99.72)
Self-employed	158 (9.50) [8.15-11.03]	26 (7.20) [4.84-10.50]
Skilled/trained	341 (20.49) [18.59-22.53]	71 (19.67) [15.77-24.23]
Unskilled	161 (9.68) [8.32-11.22]	31 (8.59) [6.00-12.09]
“White-collar”/non-manual worker	863 (51.86) [49.43-54.29]	179 (49.58) [44.32-54.86]
Housewife/house husband	21 (1.26) [0.80-1.96]	2 (0.55) [0.10-2.21]
Student	86 (5.17) [4.18-6.37]*	39 (10.80) [7.88-14.58]*
Unemployed/retired	34 (2.04) [1.44-2.88]	13 (3.60) [2.01-6.23]
Household income (before tax)	1635 (97.90)	357 (98.62)
<100,000 DKK	60 (3.67) [2.83-4.73]	15 (4.20) [2.46-6.99]
100,000 – 200,000 DKK	304 (18.59) [16.75-20.58]	57 (15.97) [12.41-20.28]
200,000 – 400,000 DKK	469 (28.69 [26.51-30.96]	87 (24.37) [20.07-29.23]
400,000 – 600,000 DKK	287 (17.55) [15.76-19.50]	64 (17.93) [14.17-22.39]
600,000 – 800,000 DKK	221 (13.52) [11.92-15.29]	63 (17.65) [13.92-22.09]
>800,000 DKK	294 (17.98) [16.17-19.95]	71 (19.89) [15.95-24.49]
Current civil status/marital status	1665 (99.70)	360 (99.45)
Married/cohabiting	1008 (60.54) [58.14-62.89]	217 (60.28) [55.00-65.33]
Unmarried	307 (18.44) [16.62-20.40]*	92 (25.56) [21.19-30.45]*
Separated/divorced	202 (12.13) [10.62-13.82]	34 (9.44) [6.72-13.06]
Widow/widower	148 (8.89) [7.59-10.38]	17 (4.72) [2.86-7.60]
Occupational physical activity	1,070 (64.07)	282 (77.90)
Mainly sedentary work	435 (40.65) [37.70-43.67]*	151 (53.55) [47.54-59.45]*
Sitting/standing, from time to time walking	395 (36.92) [34.03-39.90]*	76 (26.95) [21.94-32.60]*
Walking, from time to time lifting	215 (20.09) [17.76-22.65]	48 (17.02) [12.93-22.04]
Heavy physical work/manual work	25 (2.34) [1.55-3.48]	7 (2.48) [1.09-5.27]
Leisure time physical activity	1655 (99.10)	360 (99.45)
Being almost completely inactive or active in light physical activity for <2 h/week	104 (6.28) [5.19-7.59]	19 (5.28) [3.29-8.26]
Light physical activity for 2-4 h/week	665 (40.18) [37.81-42.59]	137 (38.06) [33.06-43.31]
Light physical activity for >4 h/week or more strenuous physical activity 2-4 h/week	784 (47.37) [44.94-49.81]	163 (45.28) [40.08-50.58]
More strenuous physical activity for >4 h/week or regular hard physical exercise and possibly competitions several times a week	102 (6.16) [5.08-7.46]*	41 (11.39) [8.39-15.24]*
Self-rated fitness compared to peers	1664 (99.64)	360 (99.45)
Same	793 (47.66) [45.23-50.09]	168 (46.67) [41.44-51.97]
Better	612 (36.78) [34.47-39.15]	135 (37.50) [32.52-42.75]
Worse	259 (15.56) [13.87-17.42]	57 (15.83) [12.30-20.11]

Table A2.2. (Continued)

Smoking status	1639 (98.14)	358 (98.90)
Current smoker	286 (17.45) [15.66-19.39]	61 (17.04) [13.37-21.43]
Previous smoker	729 (44.48) [42.06-46.92]*	125 (34.92) [30.03-40.13]*
Non-smoker	624 (38.07) [35.72-40.48]*	172 (48.04) [42.78-53.35]*
Systolic blood pressure	1656 (99.16)	359 (99.17)
Median (mmHg)	135.00 (134.00-136.50)*	129.50 (127.0-131.50)*
Blood pressure classification	1656 (99.16)	359 (99.17)
Normal	945 (57.07) [54.64-59.46]*	248 (69.08) [63.98-73.77]*
Grade 1 hypertension	567 (34.24) [31.96-33.59]*	95 (26.46) [22.04-31.40]*
Grade 2 hypertension	112 (6.76) [5.62-8.10]	14 (3.90) [2.23-6.61]
Grade 3 hypertension	32 (1.93) [1.347-2.750]	2 (0.56) [0.097-2.221]
Self-reported general health	1662 (99.52)	359 (99.17)
Excellent	139 (8.36) [7.10-9.82]	34 (9.47) [6.74-13.10]
Very good	532 (32.01) [29.78-34.32]	123 (34.26) [29.41-39.46]
Good	716 (43.08) [40.69-45.50]	141 (39.28) [34.23-44.56]
Less good	242 (14.56) [12.92-16.37]	49 (13.65) [10.36-17.74]
Poor	33 (1.986) [1.392-2.810]	12 (3.34) [1.82-5.92]

N/n, number of observations

CI, confidence interval, CIs were calculated using the Wilson score method for proportions and the normal approximation method for medians.

* indicate non-overlapping CIs between the groups.

BMI, body mass index

WHR, waist-hip-ratio

DKK, Danish krone

Blood pressure classification is based on the 2013 European Society of Hypertension/European Society of Cardiology guidelines for the management of arterial hypertension. The normal category includes high normal.
