# Supplementary material for: Time spent cycling, walking, running, standing and sedentary: a cross-sectional analysis of accelerometer-data from 1670 adults in the Copenhagen City Heart Study: Physical behaviours among 1670 Copenhageners
Source: BMC Public Health. 2019 Oct 24;19:1370. doi: 10.1186/s12889-019-7679-z (PMC6814105; doi:10.1186/s12889-019-7679-z)
Supplement: Supplementary file 3 — Additional file 3. Time spent in physical activity types, stationary behaviours and number of steps/day. Tables presenting the time spent in physical activity types, stationary behaviours and number of steps taken per day stratified by sex, age, level of education and BMI. [file 12889_2019_7679_MOESM3_ESM.pdf]

Additional file 3

Table A3.1. Time spent in physical activity types, stationary behaviours and number of steps/day among 1670 adult Copenhageners (Denmark) participating in the fifth examination of the Copenhagen City Heart Study stratified by sex

**Behaviour, min/day**	**Women**<br>*n*=958<br>Median (Q1-Q3)<br>[95% CI]	**Men**<br>*n*=712<br>Median (Q1-Q3)<br>[95% CI]	***P***
Time in bed	484.29 (451.07-521.67)<br>[479.00-488.76]	475.00 (440.00-515.52)<br>[469.29-479.17]	<0.001
Sedentary behaviour	563.82 (491.7-626.26)<br>[555.36-573.01]	595.39 (532.15-670.10)<br>[589.57-605.61]	<0.001
Standing	192.14 (154.42-236.04)<br>[187.25-196.7]	169.11 (127.60-211.11)<br>[163.20-175.15]	<0.001
Moving	70.31 (54.01-88.89)<br>[67.94-72.18]	67.10 (52.48-85.74)<br>[64.48-69.06]	0.077
Walking	84.26 (64.98-107.41)<br>[81.89-87.29]	80.16 (61.01-105.3)<br>[76.78-83.28]	0.013
Walking slow	18.05 (12.33-25.31)<br>[17.27-18.61]	22.40 (14.70-32.56)<br>[21.12-23.41]	<0.001
Walking fast	59.40 (44.00-78.65)<br>[57.45-61.17]	50.02 (36.14-68.47)<br>[47.86-52.13]	<0.001
Climbing stairs (up/down)	4.40 (2.44-6.80)<br>[4.11-4.61]	4.32 (2.24-6.8)<br>[3.88-4.66]	0.670
Number of steps/day	9482.75 (7195.77-12179.52)<br>[9213.33-9900.33]	8952.75 (6657.55-11720.79)<br>[8581.00-9378.67]	0.002
LIPA	90.31 (70.37-112.33)<br>[87.44-93.43]	92.25 (71.60-115.92)<br>[88.85-95.05]	0.140
Cycling	1.67 (0.00-13.22)<br>[0.78-2.44]	0.69 (0.00-9.73)<br>[0.23-1.87]	0.082
Cycling, among those cycling >0 min/day<br>Prevalence	8.77 (2.50-19.18)<br>[7.73-10.23]<br>62.11%	7.54 (2.48-17.11)<br>[6.27-9.08]<br>59.13%	0.170
Running	0.11 (0.04-0.42)<br>[0.10-0.14]	0.14 (0.04-0.51)<br>[0.11-0.16]	0.110
Running, among those running >10 s/day<br>Prevalence	0.57 (0.26-3.60)<br>[0.47-0.75]<br>42.80%	0.71 (0.29-4.79)<br>[0.51-1.00]<br>45.08%	0.091
MVPA	75.66 (53.48-97.23)<br>[72.37-78.25]	65.43 (45.4-86.32)<br>[62.03-68.15]	<0.001

All estimates are in min/day except for number of steps/day that is presented in number of steps taken per day.
n, number of observations
Q1-Q3, first and third quartile
CI, confidence interval
P, p-value from Kruskal-Wallis rank sum test used to test for differences between sex.
Moving consists of small movements without regular walking during a standing posture.
Walk slow and fast corresponds to walking <100 and ≥100 steps/min, respectively.
Prevalence refers to the prevalence of cycling >0 min/day and running >10 s/day (on average), respectively.
s, seconds
LIPA, light intensity physical activity
MVPA, moderate-to-vigorous physical activity

Table A3.2. Time spent in physical activity types, stationary behaviours and number of steps/day among 1670 adult Copenhageners (Denmark) participating in the fifth examination of the Copenhagen City Heart Study stratified by age

**Behaviour, min/day**	**20-<35 yrs.** *n*=196 Median (Q1-Q3) [95% CI]	**35-<50 yrs.** *n*=250 Median (Q1-Q3) [95% CI]	**50-<65 yrs.** *n*=522 Median (Q1-Q3) [95% CI]	**65-<75 yrs.** *n*=431 Median (Q1-Q3) [95% CI]	**≥75 yrs.** *n*=271 Median (Q1-Q3) [95% CI]	***P***
Time in bed	498.25 (462.34-536.21) [486.43-506.60]	467.32 (437.63-496.28) [460.17-475.00]	471.55 (437.16-506.21) [466.00-475.83]	485.17 (452.92-524.17) [475.00-492.14]	497.50 (459.79-541.32) [489.17-509.17]	<0.001
Sedentary behaviour	571.74 (508.80-623.81) [552.50-587.59]	556.09 (485.02-619.21) [536.86-578.23]	575.32 (502.19-642.74) [561.58-583.94]	588.34 (526.30-655.78) [575.91-597.42]	598.29 (523.60-672.41) [580.00-613.96]	<0.001
Standing	171.58 (143.59-210.87) [163.36-179.71]	197.32 (161.23-235.05) [190.91-204.06]	186.45 (145.33-232.94) [180.08-193.21]	176.12 (133.81-218.16) [169.33-184.09]	175.57 (133.54-228.57) [165.15-184.00]	<0.001
Moving	60.56 (48.97-74.30) [56.64-64.36]	72.03 (58.73-90.62) [67.99-76.89]	72.23 (54.68-91.87) [69.66-74.67]	69.69 (54.06-89.20) [66.96-73.70]	63.97 (48.67-83.79) [61.66-67.18]	<0.001
Walking	81.90 (64.99-100.59) [77.87-85.67]	92.92 (75.95-117.64) [87.76-98.13]	90.40 (70.51-110.90) [87.06-94.26]	80.33 (60.29-105.5) [76.39-83.40]	62.76 (43.26-83.88) [57.84-67.09]	<0.001
Walking slow	14.24 (9.81-20.42) [13.05-16.34]	19.14 (13.57-26.78) [18.26-20.71]	20.95 (14.91-29.26) [20.11-22.31]	20.87 (14.57-31.48) [19.71-22.82]	18.38 (11.77-28.45) [16.81-20.56]	<0.001
Walking fast	59.14 (46.05-73.85) [55.47-63.02]	66.13 (51.63-82.06) [62.54-69.63]	60.27 (45.13-80.26) [58.12-64.17]	51.59 (36.40-70.86) [48.28-54.07]	38.06 (24.77-52.53) [34.75-41.46]	<0.001
Climbing stairs (up/down)	5.22 (3.75-7.69) [4.85-5.74]	5.58 (3.90-7.80) [5.19-6.08]	4.75 (2.93-7.36) [4.50-5.07]	3.56 (1.97-6.35) [3.27-3.86]	2.21 (0.90-4.10) [1.80-2.49]	<0.001
Number of steps/day	9546.83 (7661.73-11870.25) [8960.00-10168.71]	10573.08 (8737.17-13565.25) [10202.67-11239.86]	10232.33 (7841.29-12527.29) [9776.00-10510.17]	8708.5 (6521.42-11507.00) [8309.00-9176.33]	6647.29 (4587.83-9201.00) [6222.00-7200.67]	<0.001
LIPA	76.73 (61.32-93.73) [71.26-80.43]	92.66 (74.66-116.38) [89.19-98.74]	95.41 (75.3-118.59) [91.99-99.83]	95.28 (74.98-116.57) [91.68-98.88]	84.82 (63.98-108.87) [80.33-92.02]	<0.001
Cycling	13.49 (2.86-22.05) [9.63-15.83]	4.82 (0.10-15.36) [2.93-7.85]	2.45 (0.00-12.70) [0.78-3.68]	0.00 (0.00-5.38) [0.00-0.15]	0.00 (0.00-1.77) [0.00-0.00]	<0.001
Cycling, among those cycling >0 min/day<br>Prevalence	15.60 (5.57-23.69) [13.37-17.53]<br>87.24%	9.61 (2.61-17.87) [7.39-12.79]<br>77.60%	8.55 (3.10-19.16) [7.18-10.37]<br>63.60%	5.41 (1.60-12.65) [3.95-6.91]<br>49.88%	3.00 (0.68-10.43) [2.24-4.53]<br>38.38%	<0.001
Running	0.50 (0.17-4.62) [0.38-1.09]	0.34 (0.11-3.44) [0.25-0.44]	0.15 (0.05-0.45) [0.12-0.18]	0.07 (0.02-0.19) [0.06-0.08]	0.04 (0.01-0.11) [0.03-0.05]	<0.001
Running, among those running >10 s/day<br>Prevalence	1.70 (0.39-6.02) [0.98-2.87]<br>76.02%	1.32 (0.36-6.90) [0.82-2.48]<br>66.00%	0.53 (0.26-3.76) [0.36-0.80]<br>47.32%	0.46 (0.24-1.05) [0.35-0.59]<br>27.61%	0.34 (0.26-0.52) [0.28-0.45]<br>18.82%	<0.001
MVPA	82.26 (67.69-100.75) [78.59-86.77]	85.50 (69.89-106.05) [82.26-90.39]	76.53 (57.15-96.90) [72.62-79.71]	62.69 (42.98-85.01) [58.53-66.03]	43.93 (28.24-60.78) [39.30-48.42]	<0.001

All estimates are in min/day except for number of steps/day that is presented in number of steps taken per day.
yrs., years
n, number of observations
Q1-Q3, first and third quartile
CI, confidence interval
P, p-value from Kruskal-Wallis rank sum test used to test for differences between age groups.
Moving consists of small movements without regular walking during a standing posture.
Walk slow and fast corresponds to walking <100 and ≥100 steps/min, respectively.
Prevalence refers to the prevalence of cycling >0 min/day and running >10 s/day (on average), respectively.
s, seconds
LIPA, light intensity physical activity
MVPA, moderate-to-vigorous physical activity

Table A3.3. Time spent in physical activity types, stationary behaviours and number of steps/day among 1664 adult Copenhageners (Denmark) participating in the fifth examination of the Copenhagen City Heart Study stratified by level of education

**Behaviour, min/day**	**No further education** *n*=190 Median (Q1-Q3) [95% CI]	**Short education (≤3 yrs. w. books)** *n*=178 Median (Q1-Q3) [95% CI]	**Vocational education (1-3 yrs.)** *n*=423 Median (Q1-Q3) [95% CI]	**Higher education (≥3 yrs.)** *n*=419 Median (Q1-Q3) [95% CI]	**University education** *n*=454 Median (Q1-Q3) [95% CI]	***P***
Time in bed	490.25 (449.92-535.96) [478.33-501.00]	483.75 (448.38-523.92) [470.00-495.00]	476.67 (438.25-522.17) [470.83-485.00]	478.33 (449.71-513.78) [472.43-483.00]	477.75 (448.04-511.54) [473.83-485.80]	0.110
Sedentary behaviour	587.55 (531.11-655.92) [574.25-609.07]	588.64 (517.69-660.66) [563.44-607.5]	578.3 (498.39-658.97) [563.99-592.69]	566.27 (488.23-630.74) [554.66-579.14]	580.65 (522.41-637.51) [571.90-588.91]	0.010
Standing	175.8 (135.46-214.39) [164.84-184.09]	179.74 (142.99-226.68) [170.51-190.38]	185.04 (142.69-233.08) [176.22-191.66]	189.99 (145.77-232.89) [180.51-198.49]	176.68 (145.71-218.82) [171.79-184.74]	0.090
Moving	66.14 (49.67-84.71) [61.16-70.60]	65.81 (52.03-86.08) [62.78-71.74]	72.70 (55.21-92.26) [69.29-76.24]	71.17 (54.61-90.73) [68.51-73.70]	65.84 (53.14-80.84) [62.99-68.57]	<0.001
Walking	72.81 (54.32-100.48) [68.21-79.72]	76.86 (56.62-100.94) [70.46-81.84]	82.28 (59.07-107.10) [77.17-87.54]	88.61 (67.85-114.49) [84.27-93.34]	83.01 (66.80-102.24) [80.67-86.86]	<0.001
Walking slow	17.59 (11.77-29.67) [16.38-21.35]	17.70 (12-24.92) [15.73-19.08]	20.22 (13.18-29.59) [19.19-21.71]	19.77 (13.61-29.49) [18.73-20.96]	19.58 (14.12-27.03) [18.59-20.91]	0.017
Walking fast	48.29 (34.11-66.77) [44.77-52.95]	52.45 (38.57-73.42) [47.30-57.23]	54.60 (34.87-75.28) [50.71-59.04]	60.37 (44.84-79.62) [58.25-64.47]	55.26 (43.84-71.24) [52.96-57.49]	<0.001
Climbing stairs (up/down)	3.38 (1.6-5.42) [2.68-3.66]	3.76 (1.93-5.69) [3.03-4.49]	3.57 (1.90-6.01) [3.21-3.90]	4.90 (2.74-7.53) [4.59-5.29]	5.21 (3.40-7.74) [4.85-5.64]	<0.001
Number of steps/day	8324.65 (5870.12-11283.67) [7543.00-8788.86]	8566.93 (6296.58-11126.36) [7776.40-9314.17]	9169.17 (6504.62-11961.15) [8472.17-9603.83]	9993.5 (7509.17-12849.67) [9345.67-10558.5]	9501.08 (7523.38-11475.29) [9208.00-9975.50]	<0.001
LIPA	84.73 (67.16-117.85) [79.52-94.73]	87.12 (66.35-109.46) [78.86-91.68]	95.99 (72.36-118.4) [91.86-100.86]	95.04 (75.77-116.94) [90.93-97.83]	85.85 (70.32-107.63) [82.94-91.71]	<0.001
Cycling	0.00 (0.00-8.74) [0.00-0.41]	0.10 (0.00-7.06) [0.00-1.15]	0.00 (0.00-6.59) [0.00-0.15]	2.05 (0.00-11.71) [0.65-3.26]	5.53 (0.07-17.07) [4.01-7.31]	<0.001
Cycling, among those cycling >0 min/day Prevalence	10.03 (2.52-19.57) [6.95-14.96] 46.84%	6.41 (1.77-19.83) [3.87-12.71] 52.81%	6.91 (1.88-15.43) [4.71-8.28] 49.17%	7.29 (2.45-16.47) [5.54-9.82] 64.92%	9.59 (3.08-20.25) [8.41-11.93] 76.87%	0.031
Running	0.09 (0.03-0.28) [0.06-0.11]	0.10 (0.02-0.21) [0.07-0.12]	0.08 (0.02-0.28) [0.07-0.10]	0.13 (0.05-0.46) [0.11-0.17]	0.23 (0.07-1.53) [0.18-0.29]	<0.001
Running, among those running >10 s/day Prevalence	0.80 (0.30-3.53) [0.50-2.12] 33.16%	0.41 (0.21-1.08) [0.25-0.70] 34.83%	0.48 (0.27-2.42) [0.39-0.67] 35.93%	0.61 (0.27-3.95) [0.44-0.95] 45.11%	1.00 (0.31-5.60) [0.60-1.35] 57.49%	<0.001
MVPA	58.87 (39.08-84.12) [53.72-65.76]	66.42 (44.54-87.66) [59.50-71.44]	66.18 (42.52-90.47) [59.85-70.88]	77.71 (53.46-98.50) [71.89-80.61]	75.51 (59.89-94.82) [72.24-77.77]	<0.001

There were 6 missing values about level of education resulting in data on 1664 participants.
All estimates are in min/day except for number of steps/day that is presented in number of steps taken per day.
yrs., years
n, number of observations
Q1-Q3, first and third quartile
CI, confidence interval
P, p-value from Kruskal-Wallis rank sum test used to test for differences between levels of education.
Moving consists of small movements without regular walking during a standing posture.
Walk slow and fast corresponds to walking <100 and ≥100 steps/min, respectively.
Prevalence refers to the prevalence of cycling >0 min/day and running >10 s/day (on average), respectively.
s, seconds
LIPA, light intensity physical activity
MVPA, moderate-to-vigorous physical activity

Table A3.4. Time spent in physical activity types, stationary behaviours and number of steps/day in 1668 adult Copenhageners (Denmark) participating in the fifth examination of the Copenhagen City Heart Study stratified by BMI

**Behaviour, min/day**	**Normal** *n*=814 Median (Q1-Q3) [95% CI]	**Overweight** *n*=620 Median (Q1-Q3) [95% CI]	**Obese** *n*=234 Median (Q1-Q3) [95% CI]	***P***
Time in bed	477.32 (445.00-514.09) [474.17-481.33]	480.83 (445.38-528.54) [475.40-489.33]	491.67 (448.67-524.58) [475.33-499.83]	0.150
Sedentary behaviour	554.96 (486.35-616.00) [543.49-564.62]	589.97 (530.28-660.33) [584.23-596.64]	632.33 (551.96-698.99) [620.33-649.09]	<0.001
Standing	198.18 (159.31-238.08) [192.95-202.49]	173.76 (134.32-211.25) [168.49-178.19]	153.07 (113.63-193.00) [144.85-160.35]	<0.001
Moving	73.02 (58.73-92.27) [71.46-76.18]	66.83 (51.80-83.99) [64.21-68.71]	57.86 (43.84-76.94) [53.22-61.22]	<0.001
Walking	87.56 (66.86-110.69) [84.04-90.31]	80.16 (62.36-103.82) [77.20-82.30]	74.72 (52.06-100.20) [65.88-81.47]	<0.001
Walking slow	20.42 (13.71-28.7) [19.38-21.22]	19.5 (13.56-28.28) [18.52-20.71]	16.96 (10.65-24.69) [15.78-18.61]	0.001
Walking fast	58.39 (43.56-76.93) [56.42-60.44]	52.69 (39.13-72.04) [50.85-54.89]	49.11 (32.98-69.97) [44.49-54.91]	<0.001
Climbing stairs (up/down)	5.17 (3.12-7.73) [4.86-5.37]	3.82 (2.14-6.34) [3.58-4.17]	3.01 (1.59-4.60) [2.53-3.29]	<0.001
Number of steps/day	9931.48 (7442.83-12582.23) [9529.33-10202.67]	8943.33 (6838.35-11608.96) [8539.29-9315.50]	8018.10 (5650.33-10970.39) [7238.86-8731.00]	<0.001
LIPA	96.35 (77-118.17) [93.83-98.74]	88.99 (68.37-111.35) [84.79-92.14]	75.60 (57.07-100.40) [70.5-82.04]	<0.001
Cycling	3.43 (0.00-15.48) [2.54-4.94]	0.11 (0.00-7.09) [0.00-0.44]	0.00 (0.00-5.53) [0.00-0.39]	<0.001
Cycling, among those cycling >0 min/day Prevalence	10.12 (2.64-19.79) [8.77-11.39] 69.04%	6.35 (2.19-17.04) [5.26-7.70] 53.71%	6.77 (2.10-13.58) [4.06-8.65] 46.58%	0.005
Running	0.21 (0.07-1.05) [0.19-0.24]	0.09 (0.03-0.28) [0.08-0.10]	0.06 (0.02-0.15) [0.04-0.07]	<0.001
Running, among those running >10 s/day Prevalence	0.77 (0.28-5.12) [0.60-1.03] 55.04%	0.52 (0.25-3.01) [0.39-0.80] 35.80%	0.35 (0.23-0.94) [0.29-0.51] 22.65%	0.002
MVPA	78.26 (56.57-98.51) [75.28-81.28]	65.77 (46.87-88.86) [62.97-68.73]	59.65 (39.64-80.30) [54.03-64.97]	<0.001

There were 2 missing values about BMI resulting in data on 1668 participants.
All estimates are in min/day except for number of steps/day that is presented in number of steps taken per day.
BMI, body mass index; WHO's classification: underweight, <18.5 kg/m^2; normal, 18.5-<25.0 kg/m^2; overweight, 25.0-<30.0 kg/m^2; obese, ≥30 kg/m^2. The underweight category was merged with the normal weight category because of low number of underweight individuals.
n, number of observations
Q1-Q3, first and third quartile
CI, confidence interval
P, p-value from Kruskal-Wallis rank sum test used to test for differences between BMI classes.
Moving consists of small movements without regular walking during a standing posture.
Walk slow and fast corresponds to walking <100 and ≥100 steps/min, respectively.
Prevalence refers to the prevalence of cycling >0 min/day and running >10 s/day (on average), respectively.
s, seconds
LIPA, light intensity physical activity
MVPA, moderate-to-vigorous physical activity
