# Supplementary material for: Time spent cycling, walking, running, standing and sedentary: a cross-sectional analysis of accelerometer-data from 1670 adults in the Copenhagen City Heart Study: Physical behaviours among 1670 Copenhageners
Source: BMC Public Health. 2019 Oct 24;19:1370. doi: 10.1186/s12889-019-7679-z (PMC6814105; doi:10.1186/s12889-019-7679-z)
Supplement: Supplementary file 4 — Additional file 4. Prevalence of spending time above/below health-related thresholds in physical behaviours. Tables presenting the prevalence of spending time above or below health-related thresholds in physical activity types and stationary behaviours, overall and stratified by sex, age, level of education and BMI. [file 12889_2019_7679_MOESM4_ESM.pdf]

Additional file 4

Table A4.1. Prevalence of spending time above or below health-related thresholds in physical activity types and stationary behaviours in 1670 adult Copenhageners (Denmark), overall and stratified by sex

**Health-related thresholds**	**Overall**<br>*N*=1670<br>*n* (%) [95% CI]	**Women**<br>*n*=958<br>*n* (%) [95% CI]	**Men**<br>*n*=712<br>*n* (%) [95% CI]	***P***
Sedentary behaviour ≥10 h/day	680 (40.72)<br>[38.36-43.12]	333 (34.76)<br>[31.76-37.88]	347 (48.74)<br>[45.01-52.47]	<0.001
Sedentary ≥10 h/day &<br>walking fast <30 min/day	148 (8.86)<br>[7.56-10.35]	58 (6.05)<br>[4.67-7.81]	90 (12.64)<br>[10.33-15.36]	<0.001
Standing ≥4 h/day	321 (19.22)<br>[17.37-21.21]	225 (23.49)<br>[20.86-26.33]	96 (13.48)<br>[11.11-16.26]	<0.001
Walking ≥1.5 h/day	704 (42.16)<br>[39.78-44.57]	417 (43.53)<br>[40.37-46.74]	287 (40.31)<br>[36.70-44.03]	0.210
Steps/day <5000	160 (9.58)<br>[8.23-11.12]	73 (7.62)<br>[6.06-9.53]	87 (12.22)<br>[9.95-14.91]	0.002
LIPA ≥2 h/day	323 (19.34)<br>[17.49-21.34]	170 (17.75)<br>[15.41-20.35]	153 (21.49)<br>[18.56-24.73]	0.064
Walking fast ≥30 min/day	1461 (87.49)<br>[85.78-89.01]	869 (90.71)<br>[88.65-92.44]	592 (83.15)<br>[80.15-85.78]	<0.001
Cycling ≥15 min/day	329 (19.70)<br>[17.83-21.70]	207 (21.61)<br>[19.07-24.38]	122 (17.13)<br>[14.48-20.15]	0.027
Running ≥20 min/week	221 (13.23)<br>[11.66-14.97]	117 (12.21)<br>[10.24-14.49]	104 (14.61)<br>[12.14-17.47]	0.180
MVPA ≥0.5 h/day	1533 (91.80)<br>[90.35-93.05]	894 (93.32)<br>[91.50-94.78]	639 (89.75)<br>[87.23-91.83]	0.011

N/n, number of observations
%, percent of individuals above/below threshold
CI, confidence interval; calculated using *Wilson's score method*
P, p-value from Pearson's Chi-squared test with Yate's continuity correction
Time cut-points (e.g., ≥10 h/day) refer to individuals' mean daily time spent in the activity types.
Walk fast corresponds to walking ≥100 steps/min.
Running ≥20 min/week is based on a daily threshold of ≥2.86 min/day.

Table A4.2. Prevalence of spending time above or below health-related thresholds in physical activity types and stationary behaviours in 1670 adult Copenhageners (Denmark) stratified by age

**Health-related thresholds**	**20-<35 yrs.** *n*=196 *n* (%) [95% CI]	**35-<50 yrs.** *n*=250 *n* (%) [95% CI]	**50-<65 yrs.** *n*=522 *n* (%) [95% CI]	**65-<75 yrs.** *n*=431 *n* (%) [95% CI]	**≥75 yrs.** *n*=271 *n* (%) [95% CI]	***P***
Sedentary behaviour ≥10 h/day	68 (34.69) [28.14-41.86]	86 (34.40) [28.60-40.69]	205 (39.27) [35.08-43.62]	188 (43.62) [38.90-48.45]	133 (49.08) [43.00-55.18]	0.002
Sedentary ≥10 h/day & walking fast <30 min/day	6 (3.06) [1.25-6.85]	5 (2.00) [0.74-0.49]	19 (3.64) [2.27-5.73]	52 (12.06) [9.22-15.61]	66 (24.35) [19.46-29.99]	<0.001
Standing ≥4 h/day	28 (14.29) [9.86-20.16]	58 (23.20) [18.22-29.03]	113 (21.65) [18.24-25.48]	61 (14.15) [11.07-17.89]	61 (22.51) [17.77-28.04]	0.002
Walking ≥1.5 h/day	73 (37.24) [30.54-44.46]	133 (53.20) [46.81-59.49]	266 (50.96) [46.58-55.32]	170 (39.44) [34.83-44.25]	62 (22.88) [18.11-28.43]	<0.001
Steps/day <5000	9 (4.59) [2.26-8.82]	3 (1.20) [0.31-3.76]	21 (4.02) [2.57-6.19]	46 (10.67) [8.00-14.07]	81 (29.89) [24.58-35.78]	<0.001
LIPA ≥2 h/day	7 (3.57) [1.57-7.52]	51 (20.40) [15.69-26.04]	124 (23.75) [20.21-27.69]	94 (21.81) [18.06-26.07]	47 (17.34) [13.14-22.50]	<0.001
Walking fast ≥30 min/day	188 (95.92) [91.83-98.09]	244 (97.60) [94.60-99.02]	496 (95.02) [92.69-96.66]	363 (84.22) [80.36-87.46]	170 (62.73) [56.65-68.45]	<0.001
Cycling ≥15 min/day	90 (45.92) [38.84-53.16]	64 (25.60) [20.40-31.56]	113 (21.65) [18.24-25.48]	43 (9.98) [7.39-13.30]	19 (7.01) [4.39-10.91]	<0.001
Running ≥20 min/week	63 (32.14) [25.77-39.23]	69 (27.60) [22.24-33.66]	73 (13.98) [11.19-17.33]	15 (3.48) [2.03-5.80]	1 (0.37) [0.02-2.36]	<0.001
MVPA ≥0.5 h/day	194 (98.98) [95.97-99.82]	249 (99.60) [97.44-99.98]	510 (97.70) [95.91-98.75]	389 (90.26) [86.96-92.81]	191 (70.48) [64.60-75.77]	<0.001

yrs., years
n, number of observations
%, percent of individuals above/below threshold
CI, confidence interval; calculated using *Wilson's score method*
P, p-value from Pearson's Chi-squared test with Yate's continuity correction
Time cut-points (e.g., ≥10 h/day) refer to individuals' mean daily time spent in the activity types.
Walk fast corresponds to walking ≥100 steps/min.
Running ≥20 min/week is based on a daily threshold of ≥2.86 min/day.

Table A4.3. Prevalence of spending time above or below health-related thresholds in physical activity types and stationary behaviours in 1664 adult Copenhageners (Denmark) stratified by level of education

**Health-related thresholds**	**No further education** *n*=190 *n* (%) [95% CI]	**Short education (≤3 yrs. w. books)** *n*=178 *n* (%) [95% CI]	**Vocational education (1-3 yrs.)** *n*=423 *n* (%) [95% CI]	**Higher education (≥3 yrs.)** *n*=419 *n* (%) [95% CI]	**University education** *n*=454 *n* (%) [95% CI]	***P***
Sedentary behaviour ≥10 h/day	86 (45.26) [38.09-52.63]	80 (44.94) [37.55-52.56]	177 (41.84) [37.12-46.72]	149 (35.56) [31.01-40.38]	185 (40.75) [36.22-45.44]	0.100
Sedentary ≥10 h/day & walking fast <30 min/day	27 (14.21) [9.73-20.18]	17 (9.55) [5.83-15.09]	57 (13.48) [10.44-17.19]	24 (5.73) [3.78-8.52]	23 (5.07) [3.31-7.62]	<0.001
Standing ≥4 h/day	30 (15.79) [11.07-21.94]	35 (19.66) [14.25-26.42]	98 (23.17) [19.29-27.54]	89 (21.24) [17.48-25.54]	68 (14.98) [11.89-18.67]	0.017
Walking ≥1.5 h/day	63 (33.16) [26.61-40.4]	64 (35.96) [29.01-43.52]	187 (44.21) [39.43-49.09]	205 (48.93) [44.05-53.82]	183 (40.31) [35.79-44.99]	0.001
Steps/day <5000	27 (14.21) [9.73-20.18]	24 (13.48) [9.00-19.6]	61 (14.42) [11.29-18.22]	23 (5.49) [3.59-8.24]	23 (5.07) [3.31-7.62]	<0.001
LIPA ≥2 h/day	43 (22.63) [17.02-29.37]	29 (16.29) [11.35-22.73]	103 (24.35) [20.39-28.78]	92 (21.96) [18.15-26.29]	55 (12.11) [9.33-15.56]	<0.001
Walking fast ≥30 min/day	153 (80.53) [74.03-85.76]	155 (87.08) [81.04-91.46]	340 (80.38) [76.2-83.99]	386 (92.12) [89.01-94.44]	423 (93.17) [90.34-95.24]	<0.001
Cycling ≥15 min/day	34 (17.89) [12.87-24.25]	32 (17.98) [12.79-24.58]	53 (12.53) [9.60-16.16]	77 (18.38) [14.85-22.49]	131 (28.85) [24.77-33.30]	<0.001
Running ≥20 min/week	20 (10.53) [6.71-16.00]	10 (5.62) [2.88-10.38]	37 (8.75) [6.31-11.96]	56 (13.37) [10.33-17.09]	97 (21.37) [17.74-25.48]	<0.001
MVPA ≥0.5 h/day	163 (85.79) [79.82-90.27]	164 (92.13) [86.90-95.47]	364 (86.05) [82.30-89.14]	400 (95.47) [92.88-97.17]	437 (96.26) [93.95-97.73]	<0.001

There were 6 missing values resulting in data about level of education on 1664 participants.
Short education corresponds to ≤3 years with books, vocational education to 1-3 years, higher education to ≥3 years and university education to ≥5 years.
yrs., years
n, number of observations
%, percent of individuals above/below threshold
CI, confidence interval; calculated using *Wilson's score method*
P, p-value from Pearson's chi-squared test with Yate's continuity correction
Time cut-points (e.g., ≥10 h/day) refer to individuals' mean daily time spent in the activity types.
Walk fast corresponds to walking ≥100 steps/min.
Running ≥20 min/week is based on a daily threshold of ≥2.86 min/day.

Table A4.4. Prevalence of spending time above or below health-related thresholds in physical activity types and stationary behaviours in 1668 adult Copenhageners (Denmark) stratified by BMI

**Health-related thresholds**	**Normal**<br>*n*=814<br>*n* (%) [95% CI]	**Overweight**<br>*n*=620<br>*n* (%) [95% CI]	**Obese**<br>*n*=234<br>*n* (%) [95% CI]	***P***
Sedentary behaviour ≥10 h/day	250 (30.71)<br>[27.58-34.03]	283 (45.65)<br>[41.68-49.66]	146 (62.39)<br>[55.82-68.56]	<0.001
Sedentary ≥10 h/day &<br>walking fast <30 min/day	49 (6.02)<br>[4.53-7.94]	62 (10.00)<br>[7.81-12.70]	37 (15.81)<br>[11.51-21.27]	<0.001
Standing ≥4 h/day	198 (24.32)<br>[21.44-27.45]	98 (15.81)<br>[13.07-18.97]	24 (10.26)<br>[6.81-15.05]	<0.001
Walking ≥1.5 h/day	382 (46.93)<br>[43.46-50.42]	242 (39.03)<br>[35.19-43.01]	79 (33.76)<br>[27.81-40.26]	<0.001
Steps/day <5000	58 (7.13)<br>[5.50-9.17]	58 (9.35)<br>[7.23-11.99]	44 (18.80)<br>[14.13-24.53]	<0.001
LIPA ≥2 h/day	189 (23.22)<br>[20.39-26.3]	106 (17.10)<br>[14.26-20.35]	28 (11.97)<br>[8.23-16.99]	<0.001
Walking fast ≥30 min/day	731 (89.80)<br>[87.47-91.75]	543 (87.58)<br>[84.66-90.02]	185 (79.06)<br>[73.17-83.97]	<0.001
Cycling ≥15 min/day	212 (26.04)<br>[23.09-29.23]	91 (14.68)<br>[12.04-17.77]	25 (10.68)<br>[7.17-15.54]	<0.001
Running ≥20 min/week	155 (19.04)<br>[16.43-21.95]	60 (9.68)<br>[7.52-12.35]	6 (2.56)<br>[1.05-5.76]	<0.001
MVPA ≥0.5 h/day	759 (93.24)<br>[91.24-94.83]	571 (92.10)<br>[89.61-94.04]	201 (85.90)<br>[80.62-89.96]	0.001

There were 2 missing values resulting in data about BMI on 1668 participants.
BMI, body mass index; WHO's classification: underweight, <18.5 kg/m^2; normal, 18.5-<25.0 kg/m^2; overweight, 25.0-<30.0 kg/m^2; obese, ≥30 kg/m^2. The underweight category was merged with the normal weight category because of low number of underweight individuals.
n, number of observations
%, percent of individuals above/below threshold
CI, confidence interval; calculated using *Wilson's score method*
P, p-value from Pearson's chi-squared test with Yate's continuity correction
Time cut-points (e.g., ≥10 h/day) refer to individuals' mean daily time spent in the activity types.
Walk fast corresponds to walking ≥100 steps/min.
Running ≥20 min/week is based on a daily threshold of ≥2.86 min/day.
